# Complete Genome Sequences of a Diverse Group of 13 Propionibacterium acnes Bacteriophages Isolated from Urban Raw Sewage

**DOI:** 10.1128/genomeA.00224-18

**Published:** 2018-06-28

**Authors:** Gustavo Ybazeta, Jenna Graham, Jelena Trifkovic, Lyne Giroux, Mazen Saleh, Syed A. Sattar, Reza Nokhbeh

**Affiliations:** a Health Sciences North Research Institute, Sudbury, Ontario, Canada; b Department of Biochemistry, Microbiology and Immunology, Faculty of Medicine, University of Ottawa, Ottawa, Ontario, Canada; c Northern Ontario School of Medicine, Sudbury, Ontario, Canada; d CREM Co. Laboratories, Mississauga, Ontario, Canada; e Department of Biology, Faculty of Science, Laurentian University, Sudbury, Ontario, Canada

## Abstract

We present complete genome sequences of 13 Propionibacterium acnes phages isolated from urban raw sewage. They belong to the family *Siphoviridae*, have genome sizes of 29,450.6 ± 256.5 nucleotides and G+C contents of 54.14% ± 0.22% and contain 42 to 45 coding DNA sequences (CDS). Genomic sequences of 9 of 13 phages were divergent by 6 to 10%, distinguishing them as species.

## Abstract (precis)

We present complete genome sequences of 13 Propionibacterium acnes phages isolated from urban raw sewage. They belong to the family *Siphoviridae*, have genome sizes of 29,450.6 ± 256.5 nucleotides and G+C contents of 54.14% ± 0.22% and contain 42 to 45 coding DNA sequences (CDS).

## GENOME ANNOUNCEMENT

Restricted diversity among phages isolated from limited sources cannot represent the actual global diversity (1). Raw sewage from metropolitan areas with multiethnic communities is a rich and unique source of phages, reflecting the global diversity of their human populations. Such a source is crucial for obtaining a diverse collection of phages against a given pathogen for use in cocktails effective in clinical practice.

For this study, we sampled urban raw sewage every 1 to 2 months over a 6-month period from two sewage treatment facilities (Ottawa and Gatineau, Canada) and successfully obtained a genetically diverse population of phages against eight Propionibacterium acnes indicator strains.

The phage plaques, isolated by the agar overlay method, were purified and used for genomic DNA isolation. Our initial BamHI restriction analysis of the phages’ genomic DNA revealed multiple restriction banding patterns indicative of genetic diversity (not shown), which prompted further analysis by full-genome sequencing. The genomes of 13 representative Propionibacterium acnes phages from a larger collection were sequenced using Illumina HiSeq 2500 (with a paired-end 125-bp read length) and Nanopore MinION MK1 platforms. *De novo* hybrid assembly of genomes was performed using SPAdes v3.20.1 (2) and Unicycler v0.4.3 (3) as previously described (4). SeqKit v0.4.5, Sickle v1.33, and FastQC v0.11.5 were used for downsampling the Illumina reads to ∼100×, trimming reads, and quality controlling, respectively (5–7). The MinION reads were demultiplexed by Metrichor, and Porechop v0.2.1 was used to remove the adapters (8). The MinION long reads were used to produce bridges in hybrid assembly using Illumina output to generate single contigs (4, 9).

The assembled single contigs for each phage were further analyzed for accuracy and quality using Bandage v0.8.1 (10) and annotated using Rapid Annotations using Subsystems Technology (RAST) v2.0 (11). The phages are *cos* type with 11-nucleotide 3ʹ-overhang sequences at either end. They have an average genome size of 29,450.6 ± 256.5 nucleotides (Table 1) with an average G+C content of 54.14% ± 0.22% and 42 to 45 CDS.

**TABLE 1:** Summary of the phage genomes deposited in GenBank and their accession numbers

Accession no.	Phage name	Length (bp)	Avg coverage (×)	SD
MG820632	pa310	29,508	276.29	16.62
MG820633	pa59	29,507	267.26	16.35
MG820634	pa27	29,593	607.95	24.66
MG820635	pa29399-1-D_1	29,370	379.04	19.47
MG820636	pa29399-1-D_2	28,892	567.77	20.99
MG820637	pa63	29,446	691.46	26.30
MG820638	pa6919-4	29,784	241.80	15.55
MG820639	pa9-6919-4	29,784	686.45	26.20
MG820640	pa15	29,309	738.33	27.17
MG820641	pa615	29,307	619.74	24.89
MG820642	pa28	29,733	196.05	14.00
MG820643	pa35	29,491	762.90	27.62
MG820644	pa33	29,135	773.60	27.81

BLASTN analysis of these genomes against the previously published genome sequences of similar phages in the GenBank nucleotide database showed 6 to 10% variation for 9 of the 13 phages. The majority of these sequence differences were mapped to the right arm coding strand for nonstructural proteins, highlighting significant diversity among the isolates (12).

The International Committee on Taxonomy of Viruses (ICTV) defines “species” on the basis of >5% differences between two genomes (13; see also http://www.ictv.global/proposals-16/2016.034a-dB.A.v1.Pa6virus.pdf). Based on this criterion, 9 of the 13 phage genomes described here are considered distinct species. These include pa310 and pa59, pa6919-4 and pa9-6919-4, pa15 and pa615, pa33 and pa35, pa27, pa29399-1-D_1, pa29399-1-D_2, pa63, and pa28. In conclusion, successful isolation of a diverse group of phages using raw sewage from multiethnic urban centers, together with the use of multiple bacterial indicator strains, highlights the importance of the classical methods of constructing phage collections. This has important implications for strategizing development of phage cocktails for clinical use.

### Accession number(s).

The GenBank accession numbers of these phages are listed in Table 1.
